# Ectopic intrapulmonary thyroid masquerading as metastatic carcinoma of the lung: a rare case scenario

**DOI:** 10.1186/s12887-023-04003-3

**Published:** 2023-04-18

**Authors:** Yuejian Zhuo, Han Yu, Xingjian Zhou, Dongdong Zhang

**Affiliations:** 1 Department of Oncology, People’s Hospital, Hubei University of Medicine, Xiangyang No. 1, Jiefang Road No.15, Xiangyang, Hubei 441000 China; 2 Department of Pathology, People’s Hospital, Hubei University of Medicine, Xiangyang No.1, Xiangyang, 441000 China; 3 Department of endocrinology, People’s Hospital, Hubei University of Medicine, Xiangyang No. 1, Xiangyang, 441000 China

**Keywords:** Intrapulmonary ectopic thyroid gland, Intrapulmonary nodules, Metastatic cancer, Nodular goiter

## Abstract

**Background:**

The intrapulmonary ectopic thyroid gland is exceedingly rare since the ectopic thyroid was discovered. Only eight cases have been reported in the worldwide literature. We present a case of multiple intrapulmonary ectopic thyroid glands with nodular goiter in a 10-year-old girl.

**Case presentation:**

The girl was found with multiple intrapulmonary nodules in bilateral lungs during the treatment of nodular goiter. The intrapulmonary lesions were initially thought to be a high possibility of metastatic cancer. A computed tomography-guided percutaneous lung biopsy was performed, and the pathological examination confirmed that the diagnosis was ectopic intrapulmonary thyroid.

**Conclusion:**

The ectopic intrapulmonary thyroid should be considered when children with nodular goiter presenting with suspected metastases in the lung.

**Supplementary Information:**

The online version contains supplementary material available at 10.1186/s12887-023-04003-3.

## Introduction

Ectopic thyroid gland (ETG) refers to the thyroid tissue present outside the normal location of the anterior neck region and is usually due to the abnormal migration of the thyroid gland during embryonic development [1]. ETG has an incidence of 1/100,000–1/300,000 in the normal population and 1/4000–1/8000 in the population with thyroid disorder [2]. It can occur at any age, with a predilection for the 30- to 50-year-olds; 65–80% of patients are female. ETG primarily occurs at the base of the tongue, around the course of the thyroglossal duct, or laterally in the neck. Less than 10% of cases are found in the periphery, including heart, lung, adrenal, duodenum, pancreas, intestine, and other regions of the body [3]. A majority of patients with ETG are usually asymptomatic and discovered incidentally. Local compressions caused by the ectopic nodule enlargement and the associated endocrine dysfunction are the main clinical presentations in symptomatic ETG. Generally, surgery, radiofrequency ablation, radioactive iodine ablation, and exogenous thyroid hormone treatment can be considered for treating symptomatic ETG.

Among the peripheral locations where ectopic thyroid occurs, the lung is a relatively peculiar site, and only a few cases have been reported so far. Moreover, among the cases of ectopic intrapulmonary thyroid, most ectopic nodules were in a single pulmonary lobe or homolateral lobes. ETG of the bilateral lung was rarely reported. In this study, we presented a case with ETG in multiple lobes in both lungs masquerading as metastatic lung carcinoma. Also, the pertinent literature was reviewed.

## Case presentation

**Fig. 1:**
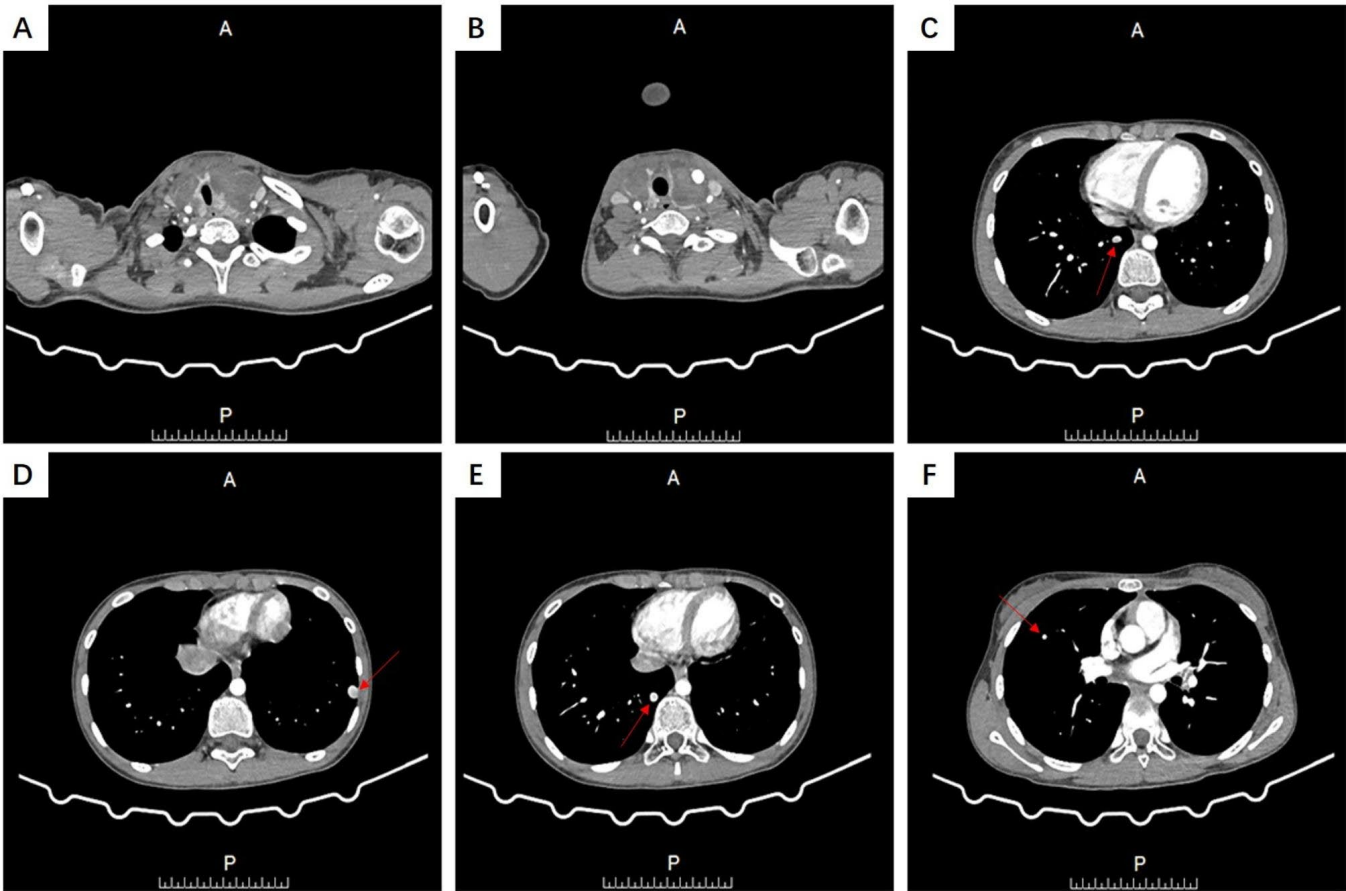
The enhanced computed tomography (CT) scanning showed abnormalities in thyroid and lung. (A-B) Multiple nodules in thyroid. (C-F) Multiple nodules in both lungs

**Fig. 2:**
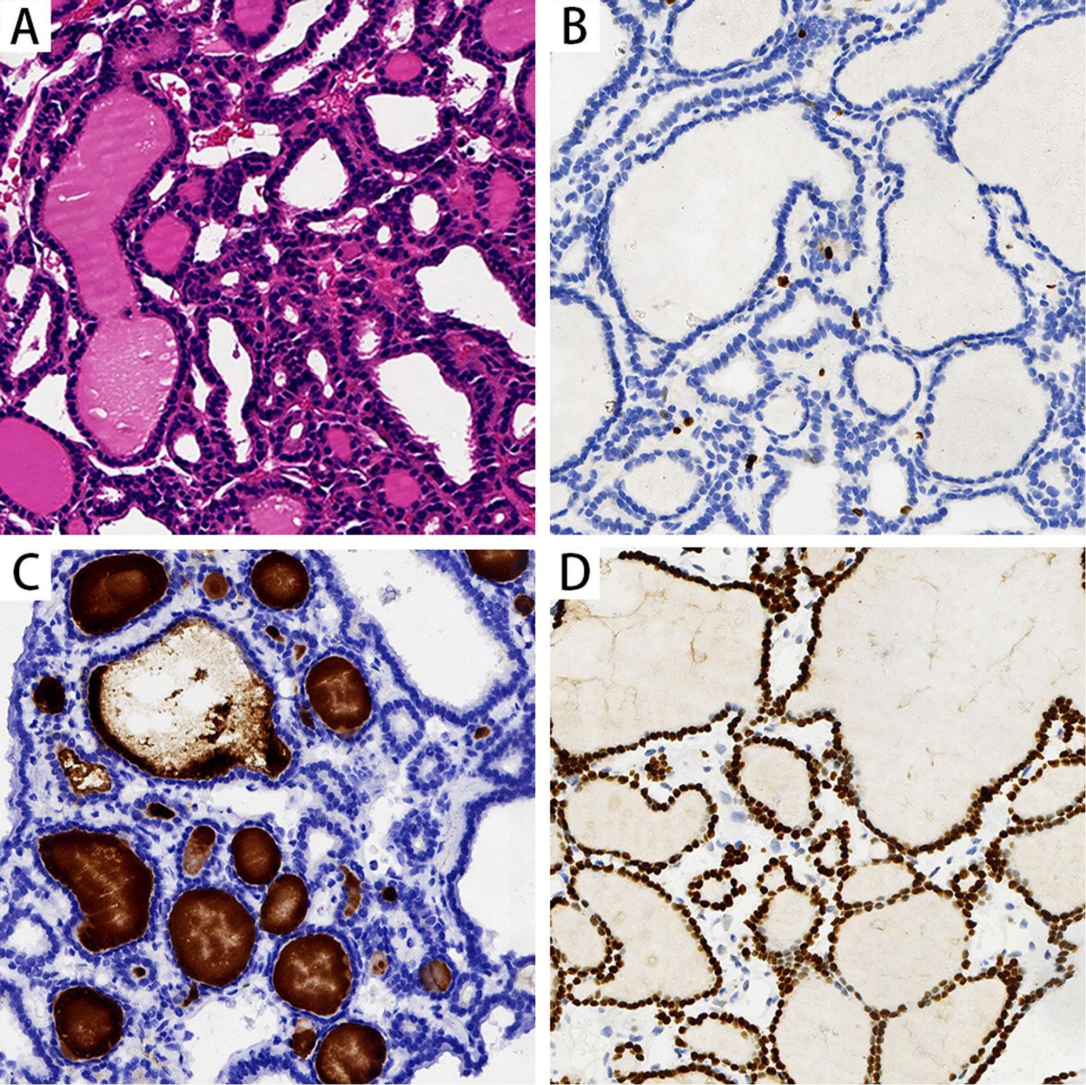
Microphotograph of pulmonary ectopic thyroid glands. (A) H&E show thyroid tissue in the pulmonary parenchyma, magnification ×200. (B) The immunohistochemistry staining of follicular epithelial cells showed low level of proliferation of Ki67, magnification ×200 (C) Positive immunohistochemistry staining for thyroglobulin (*TG*), magnification ×200. (D) Positive immunohistochemistry staining for transcription factor-1 (*TTF1*), magnification ×200

**Fig. 3:**
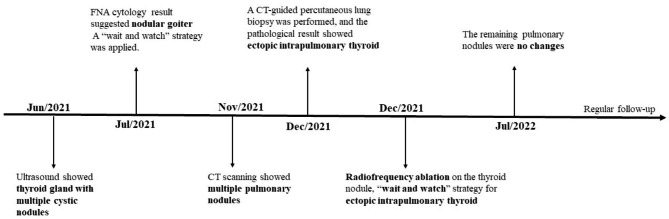
Treatment timeline of the patient

A 10-year-old girl came to Xiangyang No. 1 hospital in June 2021 due to the painless masses in the bilateral neck, which slowly increased in size in 1 year. The patient had no relevant clinical history. The ultrasound showed an enlarged thyroid gland with multiple cystic nodules in both lobes, according to the Thyroid Imaging Reporting and Data System Grade 3. The largest one was about 1.9 × 1.5 cm^2^ on the left lobe, and another one was 1.6 × 1.5 cm^2^ on the right lobe. The thyroid function test indicated that serum thyroid-stimulating hormone (TSH), free triiodothyronine (FT3), free thyroxine (FT4), and calcitonin levels were normal. But the thyroglobulin (TG) level raised to 441.4 ng/ml (normal range 0.2-70ng/ml). Ultrasonography-guided fine-needle aspiration(FNA) cytology of the thyroid gland was performed to confirm the diagnosis. The cytology results suggested nodular goiter. Radiofrequency ablation on the thyroid nodules was conducted for the main treatment. However, the routine preoperative enhanced chest computed tomography (CT) scanning showed a total of 4 nodules in both lungs, varying in size from 3 to 11 mm, with marked enhancement in the arterial phase and regression in the delayed phase (Fig. 1, **Supplementary Fig. 1**). The largest nodule was approximately 7.2 × 11 mm^2^ in size. The aforementioned imaging findings suggested metastatic carcinoma of both lungs. We performed a CT-guided lung biopsy of one nodule in each lung to define the nature of the metastases. The pathological examination showed lung tissue and well-differentiated thyroid follicular tissue, without cellular atypia. The immunohistochemistry staining showed thyroid transcription factor-1 (TTF1) and paired box gene 8 (Pax-8) positivity, thyroglobulin (TG), Cytokeratin 7 (CK7), and Cytokeratin 19 (CK19) partial positivity, and carcinoembryonic antigen (CEA), galectin 3, and Hector Battifora mesothelial-1 (HBME-1) negativity. Also, the Ki67 index suggested a low level of proliferation (Fig. 2). The pathological results of lung biopsy confirmed that the diagnosis was ectopic intrapulmonary thyroid. Considering thyroidectomy or radioactive iodine ablation might be harmful to the growth and development of the young girl, and the patient’s parents refused surgical treatment, radiofrequency ablation on the thyroid nodules was selected for the treatment of the neck mass. In view of the multiple intrapulmonary thyroid nodules in both lungs and the patient being asymptomatic, we applied the “wait and watch” strategy. One month after radiofrequency ablation, the patient is euthyroid (FT3:5.39pmol/L, FT4 :14.55pmol/L, TSH:1.02mIU/L) and has no associated complications. After the follow-up of 8 months, the CT scan showed no change in the pulmonary nodules in the bilateral lungs. Meanwhile, the patient’s serum FT3, FT4 and TSH were still in normal range, and TG level (80.72ng/ml) was nearly within the normal range (normal range 0.2-70ng/ml). The detailed diagnosis and treatment procedures are summarized in Fig. 3.

## Discussion

In normal anatomy, the thyroid is located in the anterior neck region between the second and fourth tracheal rings. During embryonic development, the developing thyroid migrates from the thyroid primordia to its final position anterior to the trachea, and when the thyroid fails to descend along the midline to reach its normal position, ETG occurs [1, 4]. The majority of ETG is located at the base of the tongue, especially in the area of the foramen cecum, which accounts for approximately 90% of reported cases.

Cases with ectopic thyroid glands in both lungs are extremely rare. Only eight cases with ectopic intrapulmonary thyroid have been reported after searching PubMed and Medline (Table 1). Most patients were asymptomatic and usually discovered incidentally or during other medical visits. A small number of patients had primary thyroid disease, including thyroid nodules, hyperthyroidism, and thyroid cancer. Some case reports indicated that primary thyroid disease might be associated with ETG, but further studies were needed to confirm this conclusion.

**Table 1:** Review of Cases of the intrapulmonary ectopic thyroid gland

Age(year)/Gender	Clinical data	Thyroid function	Lesion/Location	Size	IHC	Coexisting disease	Management	Follow-up
37/Female [5]	Neck mass	Hypothyroid	Multiple nodules Bilateral lung	3-5 mm	TG (+), TTF-1(+), CD56(+), CK19(-), HBME-1(-), Galectin-3(-)	Multinodular goiter	Total thyroidectomy and radioiodine ablation	The sizes of the remaining pulmonary nodules were reduced after 24 moths
64/Female [6]	Asymptomatic	Hypothyroid	Single nodule Left lower lobe	12 mm	NA	Thyroid cancer (After surgery)	Surgical resection	NA
83/Female [7]	Asymptomatic	Euthyroid	Single nodule Right lower lobe	25*20 mm	TG (+), Calcitonin (-)	NA	Surgical resection	NA
77/Male [8]	Asymptomatic	Euthyroid	Single nodule Left upper lobe	NA	TG (+)	Gastric cancer	NA	NA
50/Female [9]	Dysmenorrhea	Euthyroid	Multiple nodules Bilateral lung	3–7 mm	TG (+), TTF-1(+)	Endometrioid, adenocarcinoma	Surgical resection	The remaining pulmonary nodules were no changes after 6 months.
86/Female [10]	Joint pain and chest pain	hyperthyroid	Single nodule Right lung	10 cm	TG (+)	Acute myocardial infarction, hyperthyroidism, colorectal cancer	NA	NA
26/Male [11]	Asymptomatic	Unkown	Single nodule Right upper lobe	3 mm	NA	Schizophrenic psychosis occult papillary carcinoma	NA	NA
37/Female [12]	Asymptomatic	Euthyroid	Multiple nodules Bilateral lung	The maximum diameter:15 mm	TG (+), TTF-1(+),	NA	Watch and wait	The pulmonary nodules were no changes after 12 months.

Note: IHC, immunohistochemical staining; NA, not available

To date, scintigraphy using Tc-99 m, I-131, or I-123 is commonly used for diagnosing ectopic thyroid. To some extent, other imaging modalities, such as magnetic resonance imaging, CT, and ultrasound help in the complementary diagnosis [2]. Diagnosis of intrapulmonary ectopic thyroid only by imaging is difficult, because the intrapulmonary ectopic thyroid may be misdiagnosed as thymoma, germ cell tumor, neuroma, or lung metastases when viewed using CT or MRI without pathology. Positron emission tomography/computed tomography (PET/CT) can be used to excluded metastatic cancer, but it has no specificity in the diagnosis of ectopic thyroid [12]. Thus, the histopathological diagnosis is still the gold standard.

The molecular mechanism of ETG development has not been elucidated yet. Previous studies found that some genes might be related to the incidence of the disease. *Foxe1*, formerly called thyroid transcription factor-2, was involved in regulating the TG and the thyroid peroxidase gene promoters [13]. The absence of *Foxe1* in mice was associated with defective thyroid migration, which resulted in the ETG [14]. *Titf1/Nkx2-1* (thyroid transcription factor-1) is a homeodomain-containing transcription factor expressed in the human lung and thyroid gland. *Pax-8* is involved in thyroid follicular cell development and expression of thyroid-specific genes [13]. A previous study showed that Pax-8 and Titf1/Nkx2-1 interacted directly in thyroid cells and regulated the activation of the thyroglobulin promoter and differentiation of thyroid cells [15, 16]. However, currently, no evidence shows that the occurrence of ectopic thyroid is associated with these genetic mutations in humans. Further studies are needed to elucidate the mechanism.

The limitation of our study was that we did not perform a molecular biological examination, including the status of *PAX8, Titf1/Nkx2-1*, and *Foxe1* because of the limited resources. And the radioiodine imaging of the nodules in both lungs could not be performed because of the COVID-19 pandemic. Furthermore, we chose FNA rather than surgical resection for the diagnosis of the neck mass, which resulted in a lack of sufficient pathological specimens to find out the pathological difference between neck and lung lesions.

Currently, no consensus exists on the treatment of ETG. The treatment varies depending on the tumor location, size, and appearance of the clinical symptom. Based on the results of previous studies and the experience from our center, we formulated a treatment strategy that might be helpful for clinicians. For asymptomatic and euthyroid patients, a “wait and watch” strategy can be recommended [12, 17–20]. Surgery or surgical ablation is recommended when symptoms of compression or obstruction occur. Moreover, suppressive hormone therapy with levothyroxine or I-131 therapy is an alternative to surgery for patients with surgical contraindication in whom surgical resection is not possible due to anatomical difficulties [17–21].

In conclusion, we reported an exceedingly rare case of multiple bilateral pulmonary ectopic thyroid glands with nodular goiter. The patient received thyroid radiofrequency ablation treatment and regular follow-up to observe the ectopic thyroid tissues in both lungs. We think this interesting case may provoke debates about the methods of precise diagnosis and management for cases with intrapulmonary ectopic thyroid tissues.

## Electronic supplementary material

Below is the link to the electronic supplementary material.

Supplementary Material 1

Supplementary Material 2

## Data Availability

The clinical data supporting the conclusions of this manuscript are available from the corresponding author upon reasonable request.

## Abbreviations

ETG: Ectopic thyroid gland
TSH: Thyroid-stimulating hormone
TG: Thyroglobulin
FNA: Fine-needle aspiration
CT: Computed tomography
