# Supplementary figures and images for: Ectopic intrapulmonary thyroid masquerading as metastatic carcinoma of the lung: a rare case scenario
Source: BMC Pediatr. 2023 Apr 18;23:178. doi: 10.1186/s12887-023-04003-3 (PMC10110484; doi:10.1186/s12887-023-04003-3)

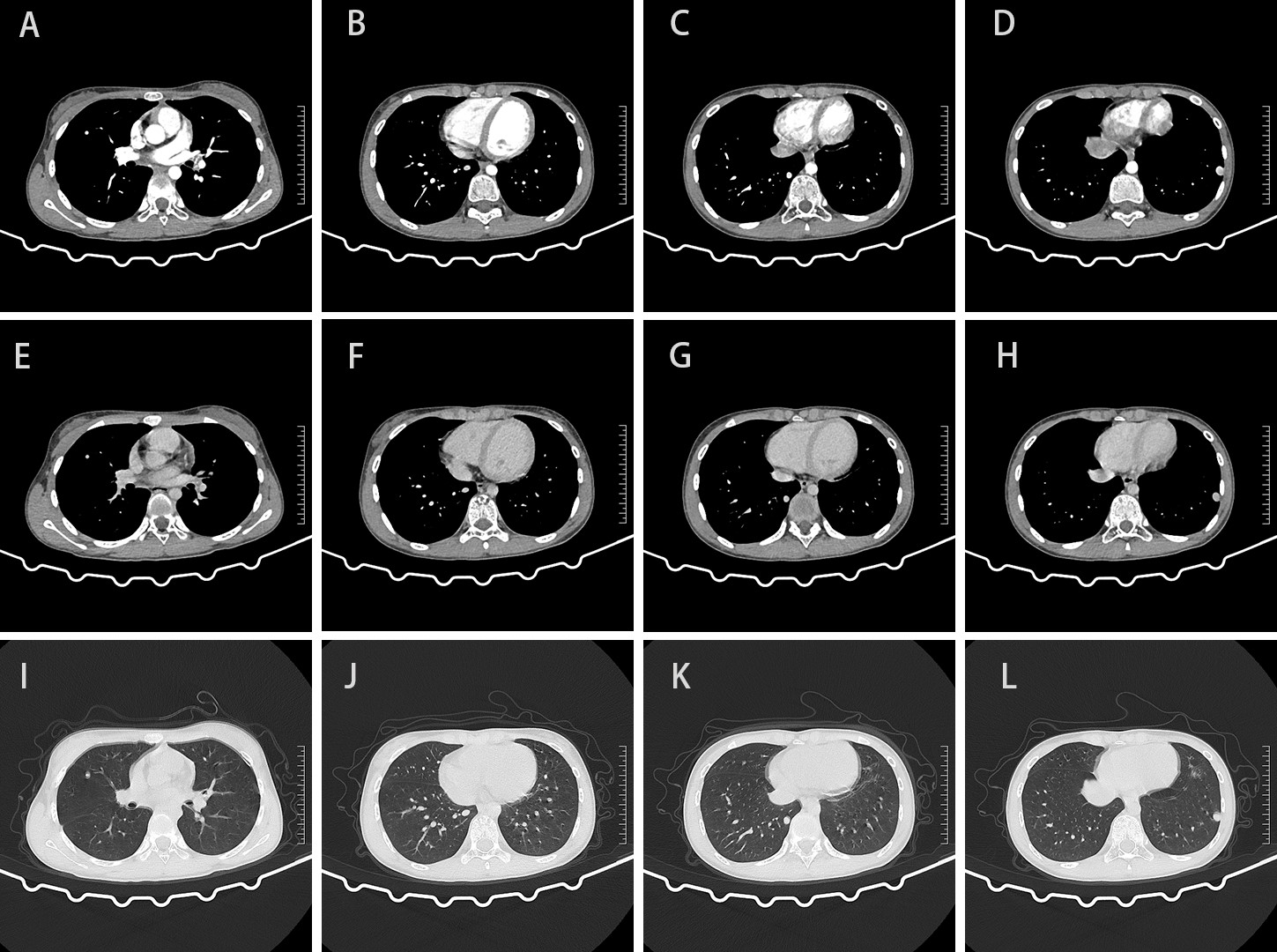

Supplement: Supplementary file 1 — Supplementary Material 1 [file 12887_2023_4003_MOESM1_ESM.jpg]
