# Supplementary material for: Ectopic intrapulmonary thyroid masquerading as metastatic carcinoma of the lung: a rare case scenario
Source: BMC Pediatr. 2023 Apr 18;23:178. doi: 10.1186/s12887-023-04003-3 (PMC10110484; doi:10.1186/s12887-023-04003-3)
Supplement: Supplementary file 2 — Supplementary Material 2 [file 12887_2023_4003_MOESM2_ESM.docx]

**Supplementary Figure 1.** The lung window and different phases images of enhanced computed tomography (CT) scanning. (A-D) The images of arterial phase. (E-H) The images of delayed phase. (I-L) The images of lung window.
